# Understanding the Risk of Human Immunodeficiency Virus (HIV) Virologic Failure in the Era of Undetectable Equals Untransmittable

**DOI:** 10.1007/s10461-020-03154-z

**Published:** 2021-01-13

**Authors:** Jayleen K. L. Gunn, Wendy Patterson, Bridget J. Anderson, Carol-Ann Swain

**Affiliations:** 1 New York State Department of Health, Albany, NY USA; 2 United States Public Health Service, Washington, USA; 3 Department of Health, AIDS Institute, Bureau of HIV/AIDS Epidemiology, New York State, Corning Tower, Albany, NY USA

**Keywords:** HIV/AIDS, Viral suppression, Surveillance, Viral load

## Abstract

The “Undetectable = Untransmittable” campaign indicates that persons living with Human Immunodeficiency Virus (HIV) who maintain a suppressed viral load cannot sexually transmit the virus. However, there is little knowledge of the percent of individuals at a population level who sustain viral suppression long term. The aims of this study were to: (1) establish a baseline of persons living with diagnosed HIV who resided in New York and had consecutive suppressed viral load tests; (2) describe the risk of virologic failure among those who were consecutively suppressed; and (3) gain an understanding of the length of time between consecutive viral suppression to virologic failure. A total of 102,339 New Yorkers aged 13–90 years were living with diagnosed HIV at the beginning of 2012; 47.9% were consecutively suppressed (last two HIV viral load test results from 2010–2011 that were < 420 days apart and < 200 copies/mL). Of consecutively suppressed individuals, 54.3% maintained viral suppression for the entire study period and 33.6% experienced virologic failure during the study period. Among persons who experienced virologic failure, 82.6% did so six or more months after being consecutively suppressed. Our findings support the need for ongoing viral load monitoring, adherence support, and ongoing risk reduction messaging to prevent forward HIV transmission.

## Introduction

Viral suppression of human immunodeficiency virus (HIV) is the pillar of the Undetectable = Untransmittable (U = U) campaign, which states that persons living with diagnosed HIV (PLWDH) cannot sexually transmit the virus when they are virally suppressed [1, 2]. Based on the evidence [3–12], over 750 organizations from 60 countries have endorsed the campaign [2, 13]. In 2017, New York State (NYS) became the first state Department of Health (DOH) in the United States (US) to endorse U = U [14].

The availability of highly effective treatment regimens with fewer side effects has enabled HIV viral suppression rates in the US to rise in recent years [15]. While promising, most clinical and surveillance studies evaluate HIV viral suppression based on the last viral load (VL) test result reported [15–18]. This cross-sectional approach may overestimate the number of persons who are stably suppressed and can be misleading in regards to U = U [18].

The empirical evidence in support of U = U has focused on the individual and community level benefits of viral suppression [3–12]. There is little understanding about long term viral suppression at the population level. The aims of this study were to: (1) establish a baseline of persons living with diagnosed HIV who resided in NYS and had consecutive suppressed VL results (i.e., the last two HIV VL results < 200 copies/mL) from 2010–2011; (2) describe the risk of virologic failure among those who were consecutively suppressed; and (3) gain an understanding of the length of time between consecutive viral suppression to virologic failure.

## Methods

The data for this retrospective cohort study were extracted from the NYSDOH HIV Surveillance System. The surveillance system, by public health law and regulation, receives electronically the results of all HIV related laboratory testing conducted for individuals residing in, or receiving HIV-related care within the state; reporting exemptions exist including that federal facilities report in the spirit of cooperation. The surveillance system includes reports for all persons diagnosed and reported with AIDS in NYS since 1981 and with HIV since 2000. The data for this study encompassed seven years, 2010–2016. HIV VL results with specimen collection dates from January 1, 2010-December 31, 2011 were used to determine initial viral suppression and study entry; sustained viral suppression was evaluated from 2012 through 2016. Reported HIV VL laboratory test results were used as a proxy for HIV medical care encounters [19].

Individual level data elements extracted from the surveillance system included: HIV diagnosis date; sex assigned at birth; age as of January 1, 2012; HIV transmission risk category; race and ethnicity; and collection dates and results of HIV VL tests from 2010–2016. Only adults and adolescents (aged 13 years and older at the beginning of 2012) were included [20]. HIV transmission risk factor information was summarized according to the CDC algorithm [21]. This risk hierarchy is based on the probability of transmission as well as the prevalence of infection among persons to whom these categories apply. HIV transmission risk categorization considers only those events that occurred prior to HIV diagnosis and may not reflect current risk behaviors. Males with a history of male-to-male sexual contact (MSM) were classified as MSM regardless of an additional history of male-to-female sexual contact. Persons with a history of injection drug use (IDU) were classified as IDU, regardless of their sexual history, except for MSM, who were classified as MSM/IDU. Other HIV risk acquisition categories include blood transfusion, perinatal exposure, and risk factor not reported or not identified. Persons’ were categorized by race and ethnicity as non-Hispanic white, non-Hispanic black, Hispanic; for this analysis, individuals categorized as non-Hispanic multi-race, non-Hispanic Native American, and non-Hispanic Asian/Pacific Islander were grouped in a category designated as other races.

Virologic failure was defined as an HIV VL ≥ 200 copies/ml. The number of days from the last suppressed VL to the first failure was calculated. To be inclusive of individuals with non-routine HIV care (i.e., minimal reported VL results), participants were censored if there was a gap of ≥ 420 days between VL test results. Participants were also censored at death or at study end (i.e., December 31, 2016) if viral suppression was sustained throughout the study period. Univariate and multivariable Cox proportional hazards were calculated to examine the association between virologic failure and the following covariates: sex assigned at birth, race/ethnicity, age as of January 1, 2012, and mode of HIV transmission. Only individuals who met the following criteria were included in the study cohort: (1) diagnosed with HIV prior to 2010; (2) age ≥ 13 and ≤ 90 years old on January 1, 2012; (3) presumed to reside in NYS at beginning of 2012; (4) had ≥ 1 VL test result after 2011; and, (5) achieved consecutive viral suppression—defined as the results of the last two VLs from 2010–2011, < 200 copies/mL, and < 420 days apart. A secondary analysis among individuals who experienced virologic failure was performed to categorize the length of time between consecutive viral suppression and virologic failure. All analyses were performed using SAS 9.4 (Cary, NC). The data reported in this study were under a comprehensive HIV surveillance IRB exemption.

## Results

In 2011, there were 102,339 PLWDH aged 13–90 years residing in NYS. PLWDH were predominately male (69.4%), aged 40–59 years (64.1%), had MSM and MSM/IDU transmission risk (38.4%), and were persons of color (non-Hispanic black 40.5%; Hispanic 32.2%). Overall, 47.9% (49,021) were consecutively virally suppressed (Table 1). Of consecutively suppressed individuals, 54.3% (26,637) maintained viral suppression for the entire study period, 33.6% (16,465) experienced virologic failure during the study period, 7.7% (3782) experienced a reported viral load gap of ≥ 420 days, and 4.4% (2137) died during the study period before experiencing virologic failure or a gap of ≥ 420 days. There were 1352 deaths among the 16,465 individuals who experienced virologic failure. The median time to virologic failure for these individuals was 362 days.

**Table 1:** Summary of study population

Subgroup	Overall population^a^		Consecutively suppressed^b^ study start		Results of consecutively suppressed population							
							Censored					
					Unsuppressed during the study		Sustained suppression throughout study		≥ 420 days between viral load tests		Death	
	n	%	n	%	n	%	n	%	N	%	n	%
Total	102,339		49,021		16,465		26,637		3,782		2,137	
Sex assigned at birth												
Male	71,029	69.41	34,048	69.46	11,015	66.90	18,763	70.44	2,729	72.16	1,541	72.11
Female	31,310	30.59	14,973	30.54	5,450	33.10	7,874	29.56	1,053	27.84	596	27.89
Age^c^ in years												
13–17	520	0.51	234	0.48	123	0.75	103	0.39	8	0.21	0	0.00
18–29	6,644	6.49	1,964	4.01	823	5.00	938	3.52	196	5.18	7	0.33
30–39	14,376	14.05	5,426	11.07	1,914	11.62	2,951	11.08	510	13.48	51	2.39
40–49	32,534	31.79	14,884	30.36	5,030	30.55	8,315	31.22	1,233	32.60	306	14.32
50–59	33,109	32.35	17,375	35.44	5,867	35.63	9,471	35.56	1,238	32.73	799	37.39
60 +	15,156	14.81	9,138	18.64	2,708	16.45	4,859	18.24	597	15.79	974	45.58
HIV transmission risk category												
Heterosexual	29,945	29.26	14,572	29.73	5,079	30.85	7,943	29.82	1,064	28.13	486	22.74
IDU	16,626	16.25	7,526	15.35	3,065	18.62	3,283	12.32	462	12.22	716	33.50
MSM + MSM/IDU	39,324	38.43	19,830	40.45	5,769	35.04	11,696	43.91	1,722	45.53	643	30.09
Unknown/Other	16,444	16.07	7,093	14.47	2,552	15.50	3,715	13.95	534	14.12	292	13.66
Race and Ethnicity												
Non-Hispanic white	20,883	20.41	11,536	23.53	2,575	15.64	7,334	27.53	1,137	30.06	490	22.93
Non-Hispanic black	41,470	40.52	17,844	36.40	6,838	41.53	8,838	33.18	1,362	36.01	806	37.72
Hispanic	32,930	32.18	15,963	32.56	5,899	35.83	8,384	31.48	1,012	26.76	668	31.26
Other	7,056	6.89	3,678	7.50	1,153	7.00	2,081	7.81	271	7.17	173	8.10

^a^Number of persons diagnosed with HIV in New York State aged 13–90 years

^b^Consecutively Suppressed: results of the last two viral loads from 2010 to 2011, < 200 copies/ml, and < 420 days apart

^c^Age as of January 1, 2012

Virologic status varied by participant characteristic. Consecutive viral suppression increased with age: 45.0% of persons aged 13–17 years achieved consecutive viral suppression compared to 60.3% of persons aged 60 + years. Roughly half of individuals with heterosexual (48.7%) and MSM (50.4%) transmission risk achieved consecutive viral suppression. More than half of non-Hispanic whites (55.2%) achieved consecutive viral suppression whereas 48.5% of Hispanics and 43.0% of non-Hispanic blacks achieved consecutive viral suppression. Among those who achieved consecutive suppression, virologic failure was highest among the youngest age categories (52.6% for persons age 13–17 years and 41.9% for persons age 18–29 years), for persons with a history of IDU (40.7%), and persons of color (38.3% non-Hispanic blacks and 37.0% for Hispanics). More than one-third of the consecutively suppressed individuals experienced virologic failure within one year of study start (17.4% within the first six months, 19.6% between 7 to 12 months) (Table 2). Approximately 20% of individuals who experienced virologic failure did so after 3 years of maintaining viral suppression. Among those experiencing virologic failure, the median time to virologic failure was 525 days (1.4 years). A shorter time to virologic failure was observed for individuals aged 18–29 years old (median = 448 days) and those with a history of IDU (median = 469 days); non-Hispanic whites (median = 560 days) experienced a longer time to virologic failure (not shown in tables) than persons of other race and ethnic groups. Most individuals (93%, n = 15,325) with virologic failure eventually achieved viral suppression during the study period; the median time to the first suppressed VL test result after virologic failure was 99 days.

**Table 2:** Time to virologic failure* among those who experienced virologic failure

Time to virologic failure	N	(%)
≤ 6 months	2858	(17.4)
> 6 months-1 year	3225	(19.6)
1 + year-2 years	4424	(26.9)
2 + years-3 years	2526	(15.3)
3 + years-4 years	1886	(11.4)
4 + years	1546	(9.4)
Total	16,465	(100%)

*Defined as ≥ 200 copies/ml

In multivariable analysis, virologic failure decreased as age increased (age 30–39: adjusted [aHR] 0.70: 95%CI 0.58–0.84; age 40–49: aHR, 0.69, 95%CI 0.58–0.84; age 50–59, aHR 0.66, 95%CI 0.55–0.80; and age 60 + aHR 0.56, 95%CI 0.47–0.68) (Table 3). Persons with a history of IDU were more likely to experience virologic failure compared to individuals with heterosexual transmission risk (aHR 1.36, 95%CI 1.30–1.43). In contrast, persons with MSM and MSM/IDU transmission risk were less likely to experience virologic failure compared to individuals with heterosexual contact transmission risk (aHR 0.92, 95%CI:0.87–0.98). Race and ethnicity were related to risk of virologic failure, with non-Hispanic blacks (aHR 1.78, 95%CI 1.69–1.86), Hispanics (aHR 1.66, 95%CI 1.58–1.74), and individuals of other races (aHR1.38, 95%CI 1.29–1.48) more likely to experience virologic failure than non-Hispanic whites. No differences were observed in time to virologic failure between males and females.

**Table 3:** Hazard ratio of virologic failure after achieving consecutive suppression

Subgroup	Univariate hazard ratio and 95% confidence interval	Multivariable hazard ratio and 95% confidence interval
Sex assigned at birth		
Male	Ref	Ref
Female	1.14 (1.10–1.18)	0.99 (0.94–1.04)
Age group^a^		
13–17	Ref	Ref
18–29	0.83 (0.69–1.00)	0.92 (0.76–1.11)
30–39	0.65 (0.54–0.78)*	0.70 (0.58–0.84)*
40–49	0.61 (0.51–0.72)*	0.69 (0.58–0.84)*
50–59	0.61 (0.51–0.72)*	0.66 (0.55–0.80)*
60 +	0.53 (0.44–0.63)*	0.56 (0.47–0.68)*
HIV transmission risk category		
Heterosexual	Ref	Ref
IDU	1.29 (1.23–1.34)*	1.36 (1.30–1.43)*
MSM + MSM/IDU	0.82 (0.80–0.85)*	0.92 (0.87–0.98)*
Unknown/Other	1.06 (1.01–1.12)*	1.05 (0.99–1.11)
Race and Ethnicity		
Non-Hispanic white	Ref	Ref
Non-Hispanic black	1.90 (1.82–1.99)*	1.78 (1.69–1.86)*
Hispanic	1.79 (1.71–1.87)*	1.66 (1.58–1.74)*
Other	1.48 (1.38–1.58)*	1.38 (1.29–1.48)*

Virologic failure ≥ 200 copies/ml

Consecutively Suppressed: results of the last two viral loads from 2010–2011, < 200 copies/ml, and < 420 days apart

*Significant relationship at p < 0.05

^a^Age as of January 1, 2012

## Discussion

This study demonstrated that about half (48%) of New Yorkers aged 13–90 years living with diagnosed HIV achieved consecutive viral suppression. Of these, more than half (54%) maintained suppression throughout the study period. One in three individuals who achieved consecutive viral suppression experienced virologic failure during study follow-up with more than one-third doing so within one year of the study start.

The proportion with virologic failure in this study was similar or higher than reported in previous research [22–25]. Researchers have cautioned that long term viral suppression may need to be defined with a sufficiently long time period to allow for true durable suppression to be observed [22]. For example, viral suppression may need to be redefined as viral suppression for at least five to six years or at minimum for a period of two or three years of ART with CD4 counts ≥ 200 cells/mm^3^, which is consistent with current viral load monitoring and treatment recommendations [22, 26].

Consistent with the HIV literature [15, 27, 28], New Yorkers in this study with a transmission risk history of IDU as well as racial and ethnic minorities were more likely to experience virologic failure while MSM and older adults were less likely to do so. This may, in part, be due to the relationship between social determinants of health and viral suppression. Studies that capture the impact of social determinants of health on viral suppression indicate that factors such as racism, stigma, lack of or poor quality health insurance, and poverty contribute to lower rates of viral suppression [22, 29, 30]. The specific impact of health insurance status on viral suppression is mixed; some studies demonstrate increases in viral suppression rates [31–33] while others have found no association [34]. Among persons with a history of IDU social determinants of health have been associated with increased rates of virologic failure [35]. They have also been identified as likely drivers of HIV outbreaks in this population [35]. Likewise, racial and ethnic minorities are more likely to experience the impacts of systemic racism leading to decreases in viral suppression rates [36–38]. Currently, the National HIV Surveillance System does not systematically collect individual-level data on social determinants of health. Therefore, we could not explore the contribution of individual-level social determinants of health on virologic failure observed in this study. Addressing social determinants of health may lead to increases in viral suppression rates, ultimately contributing to ending the HIV epidemic.

### Implications for Policy

Our findings with regard to virologic failure support the need for ongoing risk reduction activities—such as condom use and pre-exposure prophylaxis to prevent forward HIV transmission [1, 14]. Although current U = U messaging emphasizes that PLWDH should achieve and maintain an undetectable viral load for at least 6 months prior to engaging in unprotected sexual intercourse [1, 14], our results indicate that roughly one in five had an unsuppressed viral load within six months of having consecutively suppressed viral load tests and the majority had an unsuppressed viral load result between one and four years. Thus, on-going viral load monitoring by medical providers and public health officials is warranted.

## Limitations

The Prevention Access Campaign, as well as NYS’s U = U guidelines indicate that persons living with HIV who achieve viral suppression for at least six months have a negligible risk of sexually transmitting HIV [1, 14]. The present study required that the last two consecutive VL results be < 200 copies/mL with no lower time restriction between entry tests (e.g., 90 days between suppressed VLs). This may have resulted in the 34% virologic failure rate by artificially allowing more individuals to enter into the study population. However, over 80% of individuals who experienced virologic failure did so after six months of sustained viral suppression. Therefore, it is unlikely that the inclusion criteria had a strong negative effect on the proportion who experienced virologic failure. Some individuals who were censored with a gap in care ≥ 420 days may have received VL testing that was not reported to the NYSDOH HIV surveillance system. This likely represents a minimal number of test results as biannual surveys are conducted with laboratories to identify and reconcile potential gaps in HIV-related laboratory reporting. Although < 200 copies/mL is a widely accepted measure of viral suppression [17, 26], research indicates the threshold of HIV-RNA needed to transmit the virus may be between 1000 and 1500 copies/mL [10, 39]. By using ≥ 200 copies/mL to define virologic failure, our definition more closely resembles surveillance studies than transmissibility studies. Because the literature suggest that most viral blips have a mean viral load of < 50 copies/mL [40, 41], using the surveillance definition of ≥ 200 copies/mL was thought to be a reasonable approach to define viral suppression. Prolonged increases in plasma HIV VL after an extended period of suppression remain a concern for physicians and patients, as they are often a sign of treatment failure or lack of adherence to a treatment regimen. This study could be replicated using a threshold of 1000–1500 copies/mL to gain an understanding of how many people experience virologic failure at levels above a transmissibility threshold.

## Conclusion

Despite overall high levels of viral suppression, PLWDH in NYS had high rates of virologic failure, even after achieving consecutive viral suppression. These findings support U = U messaging that emphasizes the need for ongoing VL monitoring, adherence support, and engagement in risk reduction activities to prevent forward HIV transmission. Interventions to address ways to decrease virologic failure are necessary, even for PLWDH who have achieved consecutive viral suppression and maintained it for an extended period of time.

## Data Availability

Data will not be deposited.

## Code Availability

The SAS code will not be deposited but is available upon request.
